# Breaking Down the Screen: Italian Psychologists’ and Psychotherapists’ Experiences of the Therapeutic Relationship in Online Interventions during the COVID-19 Pandemic

**DOI:** 10.3390/ijerph20021037

**Published:** 2023-01-06

**Authors:** Silvia Caterina Maria Tomaino, Gian Mauro Manzoni, Giada Brotto, Sabrina Cipolletta

**Affiliations:** 1 Department of General Psychology, University of Padua, 35131 Padova, Italy; 2 Faculty of Psychology, eCampus University, 22060 Novedrate, Italy

**Keywords:** telepsychology, online intervention, attitude, online therapeutic relationship, clinical psychology, digital health, COVID-19

## Abstract

(1) Background: The COVID-19 pandemic posed new challenges to clinical practice and delineated future directions for online interventions in psychological care. The present study aimed to explore Italian psychologists’ and psychotherapists’ experiences of online interventions during the pandemic, focusing on the strategies they used to develop and maintain therapeutic relationships with their patients. (2) Methods: Between February and July 2021, 368 Italian psychologists and/or psychotherapists completed an online survey. A mixed-methods analysis was conducted, using Jamovi to analyze quantitative data and ATLAS.ti 9 to analyze qualitative data. (3) Results: Of the participants, 62% had never delivered online interventions before the pandemic; though 95.4% were delivering online interventions at the time of the survey, many reported facing technical disruptions (77.1%) and having little confidence in the online setting (45.3%). Feeling present in online sessions—facilitated by emotional attunement, active listening, and conversational spontaneity—was reported as “very important” by 93.6%. (4) Conclusions: Overall, the COVID-19 pandemic allowed a great leap forward in the use of online interventions by Italian psychologists and psychotherapists. This period of upheaval generated not only a positive change in their attitudes toward and intention to use online interventions but also revealed associated technical and relational issues that must be properly addressed.

## 1. Introduction

Online psychological interventions are feasible for various groups of patients and have clinical effects similar to those of face-to-face interventions [1]. Such interventions are also important resources for people who live in rural areas, have practical or physical limitations, or face situations that limit their accessibility to face-to-face psychological interventions [2,3,4].

However, before the COVID-19 pandemic, professionals’ attitudes toward online interventions were very heterogeneous. Some studies identified providers’ positive attitudes toward online practice [5,6], while other studies underlined their criticisms of the efficacy of online interventions and their low confidence in their own technological abilities [7,8]. Younger professionals reported greater openness to online practice than did their older colleagues [9], and professionals who used a cognitive behavioral approach reported more positive attitudes toward online practice than did their colleagues with dynamic existential approaches [10,11]. Those who prior to the COVID-19 pandemic had already integrated online interventions in their clinical practice reported positive attitudes, trust in their abilities when practicing online [12], and fewer difficulties with digital tools and in creating effective therapeutic alliances in online settings [13].

In Italy, before the COVID-19 pandemic, online interventions were poorly delivered by psychologists and psychotherapists in their everyday practice, especially due to the widespread lack of professional education on using digital tools for clinical practice, low data security and privacy, a general preference for face-to-face psychological intervention and belief that online interventions could not replace in-person sessions, providers’ low self-confidence in their own technological abilities, and significant doubts about the viability of developing true therapeutic relationships in online sessions [2].

The Italian Service of Online Psychology and the National Council of Psychology published the latest guidelines for online interventions in 2017, defining ethical and deontological rules for online practice and underlining the importance of informing the patient of the specificities of online interventions, the risks regarding data protection, and the technical disruptions that may occur [14]. Those guidelines were soon recognized as insufficient because they were not followed by any practical resource [4]. Indeed, practitioners should carefully choose proper software and hardware for their online practice, as inadequate data protection can result in potential privacy violations [15]. Appropriate tools can also reduce disruptions, such as connection problems or poor video and/or audio quality, which can negatively affect the realization of the online session and cause frustration and distrust in both psychologists and patients, affecting the therapeutic relationship [16]. With respect to the therapeutic relationship in online psychological interventions, several studies showed that many patients felt more secure to disclose in online sessions, which were reported to be less threatening and more comfortable than face-to-face sessions [17,18,19], especially for those facing social anxiety, agoraphobia, and depression [20]; eating disorders [21]; or post-traumatic stress disorder or acute stress disorder [22].

European acceptance of online interventions in psychological care is characterized by differences in digital interventions’ implementation and provision, which are caused by differences in national health authorities’ guidelines, providers’ varying levels of specific education to practice online, and the presence or absence of specifically devised tools (software and hardware) to guarantee the safe and correct delivery of digital interventions [23]. However, restrictions on meeting face-to-face imposed by efforts to mitigate the COVID-19 pandemic led to the delivery of online interventions in a broad set of health care services, including psychological care, both to continue treating existing patients and to care for new ones [24,25]. This demanded much flexibility of professionals, who had to adapt to the emergency situation quickly [26]. This forced and unexpected transition from face-to-face to online interventions was many professionals’ first experience with the use of digital tools in clinical practice [27]. The rapid transition magnified existing difficulties even for those who had already practiced online, resulting in many practical disruptions and challenges that professionals tried to overcome independently [28]. Professionals reported difficulties in several aspects of online interventions, such as practical disruptions, poor experience, lack of specific training, and obstacles to creating and maintaining a therapeutic relationship in an online session. Indeed, many professionals were worried about the potential difficulties of developing and maintaining therapeutic relationships in online settings [7]. Furthermore, during the lockdowns, the privacy needed to deliver or receive an online session was significantly limited by the domestic environment and its implications [29].

Professionals’ experience during the pandemic positively influenced their perception of online interventions as less effective than face-to-face ones: practitioners from many countries reported a positive attitude toward online interventions [30], pointing out an interest in practicing online in the future [7,9]. The experience forced on these practitioners by the COVID-19 pandemic determined a turning point in their use of digital tools, underlining the importance of moving forward from the acute phase of the pandemic to work with policymakers and practitioners to define the needs of patients and professionals and to make online interventions part of everyday clinical practice in the future [31,32].

The present online survey aimed to explore the experiences with online interventions reported by Italian psychologists and psychotherapists during the pandemic and to clarify the extent to which the involuntary aspect of the change influenced their attitudes and intent to deliver online interventions in the future. A specific focus was placed on the professionals’ experiences with digital tools (hardware and software) and their needs and doubts regarding online practice. The exploration of those areas is a prerequisite for the investigation of relational aspects, such as the therapeutic relationship and the feeling of being present in the online setting, and allowed us to build a comprehensive understanding of the experiences and strategies that Italian professionals developed to “break down the screen” that divided them and their patients.

## 2. Materials and Methods

### 2.1. Participants

Italian psychologists and psychotherapists who responded to the online survey were recruited by two methods: online advertisements published on the social media pages and websites of the National Council of Psychology and the Italian Society of Online Psychology, and snowball sampling in which the authors invited colleagues and various national schools of psychotherapy to disseminate the online survey among their students and colleagues. Participants responded to the online advertisements or were contacted via email with a presentation of the study, technical guidance for the completion of the questionnaire, and the access link. Five hundred and forty-five potential participants clicked the link; after reading the online consent form, one candidate refused consent to participate and the others ticked the “consent” box and began the online questionnaire. However, only 368 completed it (mean age 42, 89% female, average professional experience 12.4 years, SD = 10.00), and only their answers were considered in the analyses. One hundred seventy-six responses were discarded because respondents completed less than 50% of the survey. Socio-demographic descriptive statistics are reported in Table 1

### 2.2. Procedures

To improve the response rate, researchers provided an anonymous reusable link via online advertisement and email during the survey’s dissemination, which enabled participants to start and fill in the questionnaire as well as to save their progress, guaranteeing the chance to complete it at another time. Participants were asked to express their informed consent by ticking the “consent” box or the “reject” box following the description of the survey’s purposes and organization.

A self-report questionnaire was specifically designed for the present study’s web-based survey and was made available on Qualtrics (https://www.qualtrics.com) from February to July 2021. A pilot study was carried out with 16 participants in January 2021 to verify usability; researchers used specific questions to evaluate the questionnaire’s overall comprehensibility, complexity of questions, and time necessary for completion; subsequently, they addressed the issues raised and refined the survey. The final version of the questionnaire consisted of 75 items, both close-ended (55 items) and open-ended (20 items), divided into five sections as follows: socio-demographic information (11 items), attitudes and education before the COVID-19 pandemic (7 items), professional experience during the COVID-19 pandemic (15 items), user experience with online practice (24 items), and the online therapeutic relationship (18 items). Different types of response options were used, including single answer, multiple answer, open-ended answer, and Likert scales (to score respondents’ agreement with different statements). Answers to close-ended questions were mandatory, while answers to open-ended questions were mainly optional, to limit drop-out risk. The completion required approximately 20 min.

### 2.3. Analysis

To carry out the present explorative study, researchers used a mixed-methods approach combining quantitative and qualitative analysis to develop a deeper understanding of the experiences with online interventions and therapeutic relationships online that were reported by the Italian professionals involved. Statistical analyses were performed using Jamovi [33]. A logistic regression was used to test the relationships of the demographic variables (sex, age, education, and professional experience) and theoretical approaches to the use of digital tools in clinical practice before the COVID-19 pandemic. A chi-square test revealed the association between the delivery of online interventions before and during the pandemic. The non-parametric Mann–Whitney U test was used to compare aspects of the therapeutic relationships (intimacy, proximity, shared purpose, trust in the psychologist, trust in the intervention, and sense of presence experienced by the professional and patient) of participants who used digital tools before and during the pandemic to those of professionals who adopted these tools only from the pandemic’s onset (March 2020).

Using ATLAS.ti 9 [34], researchers conducted a thematic analysis [35] of textual responses to open-ended questions. Two independent researchers read the responses to become familiar with them, annotated important information, and proceeded to manually code them with the aim of identifying the presence of common patterns (or themes) in the codebook [35]. The researchers then compared their results to verify agreement or discuss disagreement. If agreement was not reached, a third researcher was consulted to grant a third independent opinion on the combined coding and to offer a solution. This process helped to increase the validity of the codification. The thematic analysis approach relies on the identification and codification of explicit themes while minimizing their interpretation. Finally, overarching themes were identified and refined.

## 3. Results

Quantitative and qualitative results are presented together to follow the structure of the survey and to support the logically consistent reporting of findings.

### 3.1. Experience before the COVID-19 Pandemic

Of the 368 respondents, 140 (38%) reported having delivered online interventions for a mean of 4 years (SD = 3.24) before the pandemic onset, and 19.8% of this group declared that they had also conducted telephone interventions for a mean of 4.92 years (SD = 6.09). By contrast, 228 respondents (62%) reported having delivered neither online nor telephone interventions before the pandemic onset. Of those who had practiced online, only 14.3% had received specific training related to online practice. Frequency data for all other items (motivations to deliver online interventions, how respondents felt while practicing online, and digital tools used and motivations to choose them) are reported in Table 2. Logistic regression did not reveal any significant associations between pre-pandemic use of online interventions and age (*p* = 0.785), years of study (by Italian regulations, 6 years for psychologists and 10 years for psychotherapists; *p* = 0.070), or years of professional experience (*p* = 0.720). However, practicing online prior to the pandemic was significantly predicted by gender (*p* = 0.021, odds ratio = 1.970): women’s likelihood of having conducted online interventions before the pandemic was nearly double that of men. Logistic regression also tested whether the use of online interventions before the pandemic was predicted by practitioners’ theoretical orientation. This variable has six levels, reflecting the five groups of theoretical orientations created by affinity on the basis of participants’ responses: (a) cognitive behavioral approaches, (b) psychoanalytic approaches, (c) systemic relational approach, (d) humanistic–constructivist approaches, (e) other approaches, and (f) no theoretical framework. The group, including participants who reported using a cognitive behavioral approach, served as the reference level, as the literature has recognized them as having more positive attitudes toward and familiarity with the use of online intervention in everyday clinical practice. No significant difference in the odds of having delivered online interventions before the pandemic emerged between the reference group and the groups using humanistic–constructivist approaches (*p* = 0.111), a systemic relational approach (*p* = 0.429), or other approaches (*p* = 0.144). However, a significant difference was found between those groups and the groups using psychoanalytic approaches (*p* = 0.013) or having no particular theoretical framework (*p* = 0.022), showing that participants in these two groups were less likely to have used online interventions before the pandemic in comparison with their colleagues using cognitive behavioral approaches.

Participants who reported having never delivered online interventions before the pandemic’s onset (62%) were asked an open-ended question about their reasons for not doing so. From their answers, thematic analysis identified three main categories: practical limitations, personal limitations, and relational limitations. These limitations are reported with their specific subthemes in Table 3.

### 3.2. Experience during the COVID-19 Pandemic

Respondents were asked whether, starting in March 2020, they had begun delivering online interventions via videoconference, e-mail, chat, or other means. Eighty-two answered “yes” (22.3%), 268 indicated “yes, and I am still using it” (72.8%), three chose “no, but I will use it in the future” (0.8%), and fifteen answered “no” (4.1%). With respect to telephone interventions, 126 answered “yes” (34.2%), 137 replied “yes, and I am still using it” (37.2%), 6 answered “no, but I will use it in the future” (1.6%), and 99 chose “no” (26.9%). Of participants who reported having delivered online interventions before the pandemic, 80% answered “yes, and I am still using it”, and 20% said “yes.” Motivations to deliver or not to deliver online interventions during the pandemic (starting from March 2020) are reported in Table 4.

Respondents were asked how many patients out of their total number of current patients habitually received online interventions: 41.1% answered “less than 20%”, and 19.4% answered “between 20% and 40%.” When asked how many patients currently receiving online interventions would suggest returning to in-person sessions as soon as the situation allowed, 18.3% answered “more than 80%”, and 39.4% said “all of them”. Frequency data for patients’ age groups, problems treated, and proportion of the provider’s total currently assisted online are reported in Table 5.

A significant association emerged between the use of online interventions before the pandemic and during the pandemic (χ^2^ (2, N = 368) = 12.7, *p* = 0.002), while whether a practitioner practiced online before the pandemic had no significant association with the motivations “I consider it a necessity for public health” (χ^2^ (1, N = 284) = 0.638, *p* = 0.425); “my patient asked for it” (χ^2^ (1, N = 284) = 0.349, *p* = 0.555); “for financial reasons” (χ^2^ (1, N = 283) = 2.71, *p* = 0.100); “I want to be present for those who are unable to attend face-to-face sessions” (χ^2^ (1, *p* = 284) = 2.45, *p* = 0.118); “I want to get involved in the actual evolution of the use of digital tools” (χ^2^ (1, N = 284) = 0.00371, *p* = 0.951); and “other” (χ^2^ (1, N = 284) = 0.0645, *p* = 0.800).

### 3.3. Attitudes toward Online Interventions

Respondents were asked to express their level of agreement with 14 statements using a five-point Likert scale (from 1 = completely disagree to 5 = completely agree). All percentages of agreement for all the statements are reported in Table 6.

A majority of the participants (202; 79.35%) who delivered online interventions during the pandemic agreed that they hoped to continue practicing online even after the pandemic’s end. By contrast, 71 (20.7%) stated that they did not want to continue practicing online. Table 7 presents professionals’ reasons to continue practicing online and their answers to the question “From your point of view, what can be done to support online interventions and professionals who use them?”.

### 3.4. User Experience with Digital Tools

**Figure 1:**
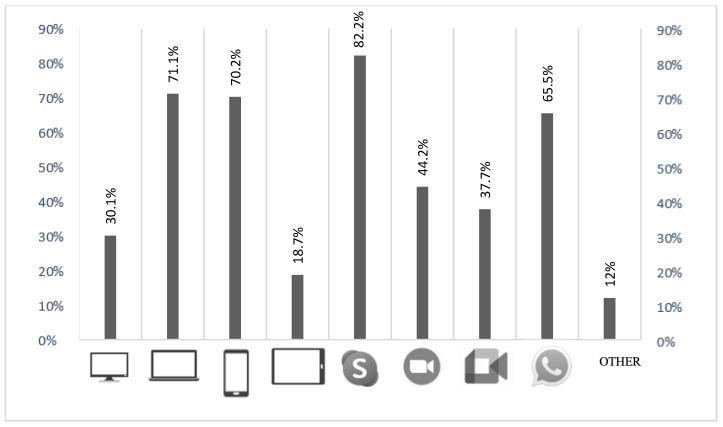
Hardware and software implemented to deliver online intervention during the pandemic.

Participants were asked to focus on the tools used to deliver online interventions. Descriptive statistics of the hardware and software used to deliver online interventions are reported in Figure 1.

When asked to rate whether the software they used was safe for online interventions, 64.3% stated “yes”, 4.7% indicated “no”, and 31% answered “I do not know.” One hundred fifty-one participants (44.2%) reported having “sometimes” faced technical disruptions during online sessions, mainly due to sound (25.7%), video (23.5%), and internet connection (77.1%) problems. Two hundred sixty-two participants (81.1%) reported having never canceled a session because of technological disruptions, and 216 (66.9%) had never received a cancellation from a patient because of technical disruptions.

Participants’ mean levels of satisfaction with hardware (M = 4.17; SD = 0.783) and software (M = 3.95; SD = 0.788) were high on a scale from 1 to 5. An intrusion and/or the occurrence of an uncontrollable event during an online session was reported by 35.6% of participants. Two hundred seventy-four (84.6%) confirmed having consulted the Italian guidelines for online practice.

### 3.5. Therapeutic Relationship Online

One hundred twelve participants (37.8%) reported that they “sometimes” perceived online sessions more tiring than face-to-face sessions, while 62 (20.9%) reported that they “often” did so. The variables that helped professionals feel connected to their patients online are reported in Table 8.

Only six professionals (2%) reported always feeling greater intimacy and emotional closeness to their patients while practicing online, while 13 (4.4%) reported that the online modality can greatly affect the trust patients have in professionals and psychological interventions. Between the therapeutic relationships of participants who reported having delivered online interventions both before and during the pandemic and those of participants who delivered them only during the pandemic, significant differences emerged on intimacy (*p* < 0.001), proximity (*p* = 0.002), and shared purpose (*p* < 0.001). The former group perceived greater intimacy, proximity, and shared purpose in their therapeutic relationships while practicing online than did their colleagues who began practicing online only in March 2020.

It was very important to feel present in online sessions to 277 participants (93.6%). Sense of presence was further explored through open-ended questions about the meaning of “being present” in the online setting and the elements that help one feel present in online sessions. Textual responses were analyzed, and two main themes were identified: “meaning of ‘being present’ online”, with seven subthemes, and “factors that help professionals and patients feel present online”, with nine subthemes. These themes and subthemes are reported in Table 9 and are illustrated by quotations chosen from participants’ responses.

## 4. Discussion

The results of the present study show that the experience of online interventions forced by the circumstances of the COVID-19 pandemic allowed Italian psychologists and psychotherapists to use and experiment with online treatment in their clinical practice, resulting in a lasting change in their attitudes toward and intent to use such tools and, thereby, charting new perspectives for the future of clinical practice. This extensive experience brought professionals challenges and disruptions but also resources and possibilities connected to the use of online interventions and revealed specific issues, such as those regarding the relational aspects and implications of the virtual setting.

### 4.1. Online Interventions in the Era of COVID-19

As reported by many studies [27,28,30,31,36], the COVID-19 pandemic provided an unprecedented opportunity to use digital tools in clinical practice for psychologists and psychotherapists all over the world. Experiences reported by professionals who delivered online interventions during the pandemic vary according to differences among countries and also across levels of “maturity” in the implementation of online interventions in the mental health care system [23].

In Italy, before the pandemic, online interventions for mental health were poorly used and mostly unintegrated in everyday clinical practice [2]; these data were confirmed by the results of the present study, showing that only 38% of participants had delivered online interventions before the first lockdown in 2020, mainly upon patients’ requests and/or due to patients’ inability to attend face-to-face sessions because of physical impediments, geographical distance (such as living in a rural area), medical conditions, or other factors. In line with previous findings [31], this study found that most participants had not delivered online interventions before the pandemic out of concern for critical issues, such as guaranteeing privacy, adequate spaces, and the management of potential software and/or hardware disruptions and its efficacy, as well as little to no demand for online interventions from their patients. Furthermore, professionals were held back from practicing online mainly by the fear of losing their professional expertise developed through in-person practice. In fact, the forced shift to the online setting during the pandemic incited many doubts and uncertainties regarding the management of the online clinical setting and its processes, strategies to develop and maintain therapeutic relationships, verbal and non-verbal communication aspects, embodiment, and more—fears that, in line with previous findings [2], were already reported by Italian professionals before the pandemic. The perceived appropriateness of the videoconference modality made it the respondents’ preferred tool for practicing online during the pandemic; as found in previous studies [37,38], this modality seemed most similar to face-to-face settings, a finding which underscores Italian professionals’ need to choose a modality that, with its specificities and variations relative to in-person consultations, guarantees practical and relational continuity of clinical practice both for professionals and for patients.

The target of online intervention changed with the pandemic: before the pandemic, the survey’s respondents worked mainly with adolescents and adults, while patients followed online during the pandemic were adults rather than children, adolescents, or older adults. This result is in line with the literature that has found specific categories of patients, such as those unfamiliar with digital tools, older adults, or children, at greater risk of practical and personal limitations in adapting to digital tools and modalities. If not adequately accounted for and addressed, such limitations could exclude those patients from receiving online interventions [9,10].

A continuity between pre-pandemic [2,9] and pandemic experience emerged in patients’ reasons for requesting online intervention: mainly relational problems and anxiety- and depression/mood-related problems. These problems worsened during the pandemic [39].

Similarly, professionals’ age, experience (in years), and degree of education (in years) did not affect the use of digital tools in clinical practice either during or before the pandemic (the latter being revealed by a prior Italian study [2]). By contrast, a study conducted in Portugal during the pandemic [9] found that younger professionals delivered online interventions more frequently than did older colleagues. Nevertheless, we have to take into account that the mean age of the present survey’s respondents was relatively high (42 years old), which may explain the absence of an effect of age on the use of digital tools in clinical practice. Furthermore, the pandemic forced a shift to the online setting, almost totally eliminating the choice of whether to practice online, an aspect that differed in previous studies of situations in which professionals who practiced online freely decided to do so.

Practitioners’ theoretical backgrounds also influenced the use of and preference for online interventions in clinical practice [30]. In the present study, participants who reported applying a psychoanalytic approach were less likely to have used online interventions before the pandemic than were participants using a cognitive behavioral approach. Previous studies have shown that professionals using cognitive behavioral or systemic relational approaches reported more positive attitudes toward online interventions than did their colleagues who used psychoanalytic or humanistic–constructivist approaches [10,11].

Almost all respondents to the present study’s survey reported delivering online interventions to their patients during the pandemic. Those who declared that they had never worked online before the pandemic’s circumstances led them to deliver online interventions to longtime as well as new patients faced many limitations, such as practical difficulties with digital tools (hardware and software), difficulty finding strategies to interact through the screen despite losing the corporeality of the interaction, and difficulties negotiating new spaces at home; these results align with those of Thome et al. [40]. These respondents’ initial attitude toward online interventions was characterized by the idea that they were not as effective as in-person sessions; however, the actual experience of online sessions changed this attitude in many such practitioners. Indeed, as was found in a previous study [9], many participants were still delivering online interventions one year after the onset of the pandemic, despite the lifting of restrictions on face-to-face meeting. Studies that were conducted before [2,10] and during the pandemic [7,41] pointed out that the direct experience of digital tools for psychological interventions resulted in providers’ development of technical and professional skills to practice online and positively influenced attitudes toward the new tools. As pointed out by Békés et al. [7], professionals who intended to continue using digital tools in the future were especially those reporting positive attitudes toward online interventions, sufficient experience with digital tools, and perceptions of those tools as effective. Most participants in the present study were willing to continue practicing online in the future because the experience they gained throughout the pandemic revealed the online setting’s convenience and usability in different situations and for various patient populations.

As was already known [16], the use of online interventions by professionals without any previous experience is often characterized by disruptions and challenges. However, the rapid, forced, and unexpected transition to the online setting at the onset of the pandemic worsened such disruptions and challenges, making the adaptation to digital tools and modalities far more difficult for both these professionals and their patients [7,42]. Respondents to the present study’s survey faced several practical, personal, and relational limitations, such as audio, video, and connection disruptions or the potential interruptions inherent to the less-private domestic setting on both sides, in line with Vallario [29]. Other challenges reported were related to providers’ concerns about ensuring privacy and security protection and feeling adequately trained to use digital tools (software and hardware) for the clinical practice, which is in line with the findings of other studies [4,43,44,45]. Moreover, professionals reported online sessions to be more tiring than those conducted in-person, especially due to the increased cognitive load imposed by interacting through a screen for extended periods and navigating difficulties in respecting personal times and private spaces [46]. This so-called “Zoom fatigue” experienced while practicing online must be explored further and properly addressed to ensure the best conditions for professionals and patients. These problems point to a need to develop and implement specific tools and educational resources to support professionals and patients in approaching, using, and proposing psychological interventions online. Participants reported asking for ad hoc digital tools (software and hardware) that would guarantee privacy and security standards while remaining accessible and easy to use for both professionals and patients, preferably provided or recommended by the National Council of Psychology. In this sense, the factors that could positively influence Italian psychologists’ and psychotherapists’ choice to deliver online psychological interventions in the future include a positive attitude toward online interventions, patients’ favorability, the experience gained during the pandemic, the provision of specifically devised tools and specific educational courses for online practice, and the clarification of ethical and deontological issues connected to online practice.

**Figure 2:**
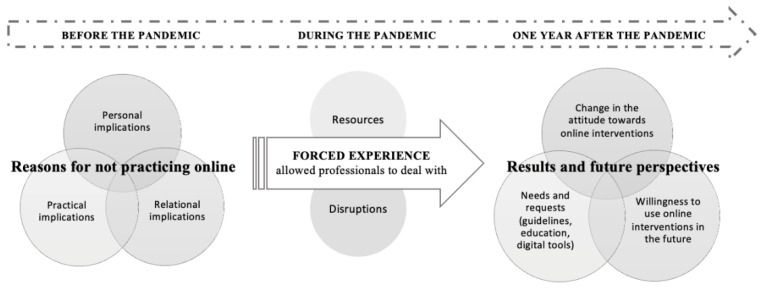
A graphic representation of the effects that the COVID-19 pandemic had on Italian psychologists’ and psychotherapists’ attitudes towards online interventions.

A comprehensive overview of this study’s results suggests that the experience of online interventions forced by the circumstances of the COVID-19 pandemic allowed a great leap forward in the use of online interventions by mental health professionals and patients all over the world [32] and, in this case, specifically for Italian professionals and patients. This evolution delineated future opportunities and directions for the use of online interventions in everyday clinical practice, as well as underlined needs and doubts regarding this practice and identified the digital tools that must be properly addressed to guarantee future progress (see Figure 2).

### 4.2. Feeling Present and Connected in the Online Setting

Practicing online magnified certain challenges of predominately in-person relationships and patient interactions, highlighting the need to define specific elements and ways to create and maintain a solid relationship while shifting from a physical to a virtual space. As the interaction is mediated by a digital tool (the screen), feeling present and connected to the other person could be challenging and demanding for both professionals and patients. Bouchard et al. [47] characterized presence in videoconference settings as comprising three aspects: feeling as though both were in the same room, being actively involved in the interaction, and feeling completely immersed in the conversation. In line with this articulation, participants reported that experiencing presence during an online session was facilitated by many aspects, such as mutual and active participation, the experience of we-ness, presence beyond space and time (as though they were in the same room), and the elaborateness of the conversation and the strength of the emotional connection. Being able to experience presence in online sessions provides participants the opportunity to create a virtual space in which self-consciousness, creativity, and collaboration can build and maintain a satisfying therapeutic relationship [36]. In line with other studies’ findings [48,49,50,51], the results of the present study’s survey show that professionals who practiced online during the pandemic were able to successfully create and maintain satisfying therapeutic relationships in online sessions, especially those who had become familiar with online interventions prior to the pandemic. Nevertheless, as in previous studies [36,52], participants identified certain factors that could support them in the online relational process, such as the availability and provision of ad hoc digital tools (e.g., software specifically devised to deliver online interventions), the implementation of national educational programs to inform and train professionals to use digital tools in their clinical practice, and the updating of specific guidelines to address doubts, correct misinformation regarding psychological practice online, and resolve ethical and deontological concerns.

**Figure 3:**
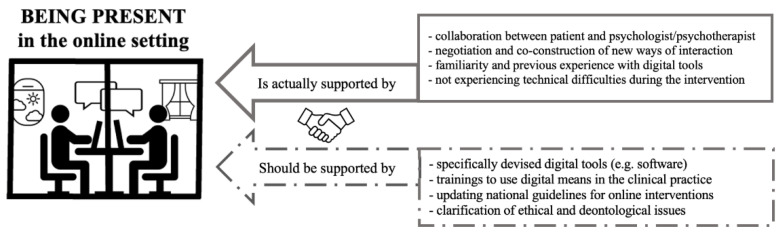
A visual representation of the factors that are involved in the experiencing of presence in the online setting and which need to be addressed to support it.

Effective therapeutic relationships in online sessions requires both professionals and patients to explore and negotiate new ways of interaction. Though these ways may be similar to those employed in an in-person session, the online setting also necessitates the collaborative construction of a dialogue and cooperation between the two parties to define opportunities as well as boundaries [18,36]. In line with this concept, our results pointed to possible future directions for interventions and further investigations, as participants underlined the importance of a multi-level collaboration among multiple parties (such as colleagues, institutions, and patients) to address the challenges and uncertainties faced by professionals who prioritize feeling present in the satisfying therapeutic relationships of their online practice (see Figure 3).

## 5. Limitations

The study has some limitations. First, the sample was composed of professionals who practice mainly in northern Italy, creating bias that prevents the generalization of results throughout Italy. Second, the survey was disseminated only online; this could have led to the recruitment of participants who were familiar with the use of digital platforms, social media, and websites and who, therefore, had a greater chance of being familiar with digital interventions or who had already used such interventions in their practice. In addition, the length of the questionnaire resulted in incomplete or interrupted submissions from many respondents, even though participants were informed of the opportunity to stop and resume the completion at another time without losing the answers already recorded. Last, the p-values reported in the result section are nominal and not corrected for multiple-hypothesis testing, as the present research reports explorative findings. Authors are aware that, for this reason, results might contain false-positive findings, underlining the importance of implementing further investigation and analyses in the future to disambiguate this possibility.

## 6. Conclusions

The COVID-19 pandemic has generated a unique experience for the Italian psychological practice, engaging mental health professionals in a forced shift from the physical room to the virtual one to continue treating patients. The necessarily abrupt adaptation to the online setting faced by professionals and patients had never before been experienced so extensively. For many, this involuntary experience and adaptation has permanently altered practitioners’ perspective on the use of digital tools in clinical practice, resulting in their experimentation with different tools and in the development of a positive attitude toward digital interventions. Professionals developed a greater confidence in their digital capabilities and a positive attitude toward the use of online interventions in the future of their everyday practice.

At one year from the onset of the pandemic, Italian professionals were accepting and exploring the implications of digital interventions, even though they continued to prefer the in-person modality. Learning from experience, Italian psychologists and/or psychotherapists have asked for specific education about the ethical and deontological aspects of practicing online, the development of specifically devised tools to deliver psychological interventions online, and increased accessibility and usability for patients, especially those who might be excluded from or have difficulty accessing treatment.

Fears regarding the development and maintenance of a satisfying therapeutic relationship online no longer appears to limit practicing online; in fact, the direct experience of the professionals involved in the study proved that being present in the online context comes with different challenges that can be overcome by adopting different strategies and negotiating new ways of interaction through the screen, accepting and facing both opportunities and disruptions.

The COVID-19 pandemic propelled the Italian context toward significant progress in the implementation of online psychological practice, which otherwise would have taken many years. Upheaval associated with the pandemic eroded professionals’ and patients’ resistance to and doubts about online treatment, making way for a new perspective on psychological intervention. This study’s overview of the Italian experience of this process allowed us to identify supports for and disruptions to online practice during such times, new questions and perspectives (such as the needs of professionals to guarantee their clinical practice online for the future), and last—but not least—the importance of exploring and accounting for the patients’ point of view and experience on the other side of the screen.

## Figures and Tables

**Table 1:** Socio-demographic descriptive statistics of the sample.

Sample Characteristics	N	%
Males	58	15.8
Females	309	83.9
Other	1	0.3
***Profession***		
Psychologists	107	29.1
Psychotherapists	261	70.9
***Psychotherapists’ theoretical orientation***		
Psychoanalytic approaches	103	39.5
Systemic relational approach	46	17.6
Cognitive behavioral approaches	56	21.4
Humanistic–constructivist approaches	36	13.8
Other approaches	20	7.7
***Professional condition***		
Self-employed	301	81.8
Working in the National Sanitary System	23	6.3
Employed in private companies	16	4.3
Employed in other fields	28	7.6
***Geographical origin***		
North (Valle d’Aosta, Liguria, Lombardia, Piemonte, Trentino-Alto Adige, Veneto, Friuli-Venezia Giulia, Emilia Romagna)	228	62
Centre (Toscana, Umbria, Marche, Lazio)	74	20.2
South (Abruzzo, Molise, Campania, Puglia, Basilicata, Calabria)	42	11.3
Islands (Sicilia, Sardegna)	24	6.5
***Age group of patients usually assisted***		
Adults		92.7
Adolescents		62.5
Children		30.2
Older people		16.6
***Main intervention areas***		
Relational problems		62.2
Anxiety disorders		60.6
Depressive and mood disorders		54.1
Family-related problems		37.8
Multiple problems		34.8
Couple-related problems		30.4
Eating disorders		19
Cognitive evaluation		13.9
Other		12.8
Problems related to work		12.5
Sexuality-related problems		12
Cognitive rehabilitation		12
Emergency-related problems (disasters or accidents)		7.9
Emergency-related problems (crisis or suicide)		7.1
Substance addiction		6.8
Internet addiction		4.9
Gambling addiction		3.3

**Table 2:** Data on those who experienced online interventions before the COVID-19 pandemic.

	%
***Reasons to deliver online interventions***	
my patient asked it	58.5
for economic reasons	0.7
I want to be present for those who are unable to attend the session in person	51.4
I want to become involved in the actual evolution of the use of digital tools	17.1
other	22.9
***How have you felt practicing online?***	
very distressed	0.7
sometimes distressed	21.4
neutral	17.1
sometimes at ease	16.4
always at ease	44.3
***Which digital tool or tools have you used?***	
e-mail	8.7
chat	7.4
videoconference	87.9
other tools	8.1
***What were the motivations to choose a specific tool?***	
personal preference	33.6
patient’s preference	30.7
the tool chosen is the most economical	5.7
the tool chosen is easy to use	25.7
the tool chosen is the most appropriate for this specific intervention	58.6
other	1.4

**Table 3:** Themes and subthemes identified analyzing the responses to the open-ended question regarding the motivations reported by the professionals who have not practiced online before the COVID-19 pandemic.

Themes	Practical Limitations	Personal Limitations	Relational Limitations
Sub-themes	1. Lack of a specific legislation	1. Personal preference for in-presence interventions	1. The online setting is detached and cold
	2. Clinical tools and techniques are not suitable for the online setting	2. Patients’ and professionals’ prejudices among the online setting	2. Lack of non-verbal communication
	3. Interventions with groups, families, and/or couples online are more complicated	3. Lack of specific education to practice online	3. Lack of spontaneity in the interaction
	4. The online setting is not easily accessible to children and older people	4. Prejudices among efficacy of online interventions	4. Loss of welcome and farewell rituals
	5. Difficulties in finding a private space at home for both professionals and patients	5. Professionals’ doubts on personal competencies when practicing online	5. Patients are less engaged and committed
	6. Patients are not used to asking for it		6. Loss of corporeity in the interaction
	7. Difficulties to manage emergency and crisis situations		7. Patients and professional are more distracted by the home setting

**Table 4:** Data on reasons whether to deliver online interventions during the COVID-19 pandemic (starting from March 2020).

Have You Delivered Online Interventions during the Pandemic?		%
	***Reasons to deliver online interventions***	
**“Yes” or**	I consider it a necessity for public health	67.8
**“Yes, and I am still using it”**	my patient asked it	40.5
	for economic reasons	8.3
	I want to be present for those who are unable to attend the session in person	57.8
	I want to become involved in the actual evolution of the use of digital tools	18.2
	other	0.7
	***Reasons not to deliver online interventions***	
**“No” or**	online interventions are not effective as those in person	33.3
**“No, but I will use it in the future”**	right now, I do not see an added value compared with those in person	20
	in general, I do not like to use technology in my clinical practice	26.7
	my patients do not want it	20
	lack of adequate software and/or hardware	6.7
	I am afraid of making mistakes	6.7
	other	33.3

**Table 5:** Data regarding patients’ assisted online at present.

***Age group of patients assisted online***	**%**
Adults	94.3
Adolescents	43.7
Children	10.9
Older people	10
***Problems treated online***	
Anxiety disorders	66.5
Relational problems	61.95
Depressive and mood disorders	51.45
Multiple problems	36.3
Family-related problems	36
Couple-related problems	20.7
Problems related to work	11.4
Eating disorders	11.2
Other	10
Sexuality-related problems	9.2
Cognitive rehabilitation	5.7
Cognitive evaluation	3.4
Substance addiction	3.1
Gambling addiction	1.4
Emergency-related problems (disasters or accidents)	1.4
Emergency-related problems (crisis or suicide)	1.4
Internet addiction	1.1
***% of patients assisted online at present***	
No one	10.6
Less than 20%	41.1
Between 20% and 40%	19.4
Between 41% and 60%	8
Between 61% and 80%	6.6
More than 80%	6
All	8.3

**Table 6:** Level of accordance with sentences on the use of online interventions.

	%	%	%	%	%
*Please Express Your Accordance with the* *Following Sentences on the Use of Online Intervention* *from 1 (Completely Disagree) to 5 (Completely Agree)*	Completely Disagree	Mostly Disagree	Neutral	Mostly Agree	Completely Agree
I am willing to use online interventions (email, chat, video) in my clinical practice	1.4	11.1	13.1	39.7	34.6
Patients have asked/are asking me for the possibility to be treated online (video, chat, email).	4.9	18.3	22.0	36.6	18.3
I feel that digital tools are useful for my clinical practice	0.6	9.4	16.0	43.1	30.9
Using the online tools (video, chat, email) facilitates my clinical practice	9.1	23.4	34.6	20.0	12.9
I feel competent in using digital tools (hardware and software)	1.7	7.4	24.3	38.0	28.6
I have attended training courses about the theory and practice of psychological interventions online (video, chat, email)	29.4	18.9	14.9	27.7	9.1
Using online interventions (video, chat, email) allows me to save time	7.4	11.4	17.1	36.3	27.7
Using online interventions (video, chat, email) allows me to save money	14.6	18.0	24.6	24.3	18.6
Using online interventions (video, chat, email) causes me to earn less	39.7	26.3	20.9	8.6	4.6
My patients think that online practice (video, chat, email) is different from the in-person one	5.4	16.0	25.4	38.6	14.6
I think that online practice (video, chat, email) does not equal the in-person one	6.3	12.9	18.0	37.7	25.1
If I could choose, I would not practice online (video, chat, email)	16.3	16.3	18.0	27.4	22.0
If I could choose, I would practice more online (video, chat, email)	29.1	31.4	19,7	11.1	8.6
I would recommend online practice to colleagues (video, chat, email)	3.1	10.9	33.4	32.0	20.6

**Table 7:** Professionals’ motivations to continue their practice online and points of view regarding ways to support the use of online interventions.

***Motivations to Continue Practicing Online after the Pandemic***	**%**
My patients find it convenient and useful	52.6
My patients ask to be assisted online	34.6
For economic reasons	8.5
For practical and convenience reasons	42.3
I got used to the online practice	15.4
I want to remain in contact with the actual evolution of the patients’ use of digital tools	21.7
Other	28.3
***What can be done to support online interventions and professionals who use it?***	
Training on hardware for the online practice	15.5
Training on software for the online practice	33.5
Education on ethical and deontological aspects of the online practice	60.3
Education on legal and normative aspects of the online practice	50.7
Education on security and privacy of the online practice	59.8
Provision of tools (hardware and software) specifically devised for the online practice	51.9
Improving the accessibility of online practice for patients	35.3
Improving the accessibility of online practice for professionals	30.9
Improving the accessibility of online practice for public services	28
Other	7

**Table 8:** Variables that help professionals to feel connected with their patients online.

*Variables That Help Professionals to Feel Connected with Their Patients Online*	%
The video quality	47
The size of the screen	23.4
The audio quality	41.6
The video angle (only face, half bust, distant, close, etc.)	38.5
The background	8.1
The emotional climate	76.7
The therapeutic relationship with the patient	88.2
Other	1.7

**Table 9:** Themes and subthemes identified analyzing the responses to the open-ended question regarding the meaning of “being present” in the online setting and the factors that help professionals and patients feel present in the online session.

Themes	*Meaning of ”Being* *Present” Online*	*Factors That Help the* *Professionals and Patients Feel Present* *Online*	*Quotations*
subthemes	1. Being there beyond space and time	1. Asking and giving feedback	*“Meta-communicating through asking constant feedback to the patient”*
	2. Experiencing we-ness	2. More sustained eye contact	*“I am present in the conversation with eye contact”*
	3. Focusing on “here and now”	3. Not getting distracted by other things	*“Trying not to get distracted and to be focused on the patients as if we were physically in my studio”*
	4. Being actively engaged with the patient	4. Losing track of time	*“I feel present when I lose track of time”*
	5. Active listening	5. Making behaviors more pronounced (e.g., explaining what I am doing)	*“Looking into the screen, explaining what I am doing (e.g., “I am taking notes”) in case it was not visible from the webcam”*
	6. Feeling an emotional attunement	6. Feeling like we are in the same room	*“I feel present when there is so much emotional attunement and empathy, that it seems that we are in the same room”*
	7. Being immersed in the conversation	7. Hearing and seeing clearly the other person	*“Video, audio, and connection have to work properly”*
		8. Spontaneity of speech turns and conversational fluency9. Feeling spontaneous while interacting	*“Spontaneity of the conversation and of the speech turns”* *“Smoothness of the conversation… as if we were dancing”*

## Data Availability

Not applicable.
